# Synthesis Monitoring, Characterization and Cleanup of Ag-Polydopamine Nanoparticles Used as Antibacterial Agents with Field-Flow Fractionation

**DOI:** 10.3390/antibiotics11030358

**Published:** 2022-03-08

**Authors:** Valentina Marassi, Sonia Casolari, Silvia Panzavolta, Francesca Bonvicini, Giovanna Angela Gentilomi, Stefano Giordani, Andrea Zattoni, Pierluigi Reschiglian, Barbara Roda

**Affiliations:** 1 Department of Chemistry G. Ciamician, University of Bologna, 40126 Bologna, Italy; sonia.casolari@unibo.it (S.C.); silvia.panzavolta@unibo.it (S.P.); stefano.giordani7@unibo.it (S.G.); andrea.zattoni@unibo.it (A.Z.); pierluigi.reschiglian@unibo.it (P.R.); barbara.roda@unibo.it (B.R.); 2 byFlow srl, 40129 Bologna, Italy; 3 Department of Pharmacy and Biotechnology, University of Bologna, 40138 Bologna, Italy; francesca.bonvicini4@unibo.it (F.B.); giovanna.gentilomi@unibo.it (G.A.G.); 4 INBB—Biostructures and Biosystems National Institute, 00136 Rome, Italy

**Keywords:** antimicrobials, synthesis monitoring, separation, flow-field flow fractionation multidetection, online characterization and purification, silver polydopamine nanoparticles, cleanup

## Abstract

Advances in nanotechnology have opened up new horizons in nanomedicine through the synthesis of new composite nanomaterials able to tackle the growing drug resistance in bacterial strains. Among these, nanosilver antimicrobials sow promise for use in the treatment of bacterial infections. The use of polydopamine (PDA) as a biocompatible carrier for nanosilver is appealing; however, the synthesis and functionalization steps used to obtain Ag-PDA nanoparticles (NPs) are complex and require time-consuming cleanup processes. Post-synthesis treatment can also hinder the stability and applicability of the material, and dry, offline characterization is time-consuming and unrepresentative of real conditions. The optimization of Ag-PDA preparation and purification together with well-defined characterization are fundamental goals for the safe development of these new nanomaterials. In this paper, we show the use of field-flow fractionation with multi-angle light scattering and spectrophotometric detection to improve the synthesis and quality control of the production of Ag-PDA NPs. An ad hoc method was able to monitor particle growth in a TLC-like fashion; characterize the species obtained; and provide purified, isolated Ag-PDA nanoparticles, which proved to be biologically active as antibacterial agents, while achieving a short analysis time and being based on the use of green, cost-effective carriers such as water.

## 1. Introduction

Advances in nanotechnology have opened up new horizons in nanomedicine through the synthesis of new nanomaterials. Among the different antibacterial approaches, metal nanoparticles (NPs) are increasingly being used as an alternative to antibiotics or in combination with them. The advantages of these strategies are numerous, including providing activity based on different and simultaneous mechanisms such as oxidative stress induction, metal ion release, and non-oxidative mechanisms; the prevention of microbial drug resistance; and their potential use for carriers of antibiotics [1]. A wide range of nanomaterials have been demonstrated to possess antimicrobial effects, including iron (III) oxide, zinc oxide, magnesium oxide, silver, gold, copper and copper oxide, calcium oxide, titanium dioxide, and cadmium oxide. Among these, silver NPs (AgNPs) show promise for use as antimicrobials able to kill both Gram-negative and Gram-positive bacteria [2] due to both their internalization in the cell due to their small size and their release of silver ions [3]. Some antibacterial properties are strictly related to particle size, with smaller particles showing improved activity due to their increased surface area to mass ratio and higher surface reaction activities [4,5,6]. Different mechanisms of ion-dependent antimicrobial action, such as interference with bacterial metabolic processes and structures, cell wall disruption, and increases in cell permeability, have been reported for AgNPs, including interaction with DNA [7] or the generation of reactive oxygen species [8,9,10,11,12,13]. Cooperative actions with antibiotics and antifungal agents are also able to reduce the need for high antibiotic dosages and therefore minimize side effects [14,15,16,17,18].

In this framework, biocompatible coatings can be used to improve antibacterial effects without increasing cytotoxicity, making the interactions with the biological system more versatile. The use of polydopamine (PDA) represents an attractive approach. PDA is a polymer consisting of dopamine, a substance that is present both in mammals as a neurotransmitter and in some molluscs as an adhesive protein. PDA can be obtained through the oxidation of dopamine or other catecholamine [19,20,21].

A recent study demonstrates that PDA coating significantly enhances the potency of PDA-AgNPs against *Escherichia coli* [19]. The interaction between Ag and the catechol group on the PDA coating is responsible for the increase in the generation of reactive oxygen species (ROS), causing bacterial damage. Ag-coated PDA microspheres have been used to kill *Staphylococcus aureus* cells due to their elevated ROS level [20].

Despite the fact that Ag-PDA particles exhibit an excellent antimicrobial activity, problems such as aggregation and toxicity limit their practical application. To solve these issues, many researchers have focused on NP preparation. In one study, PDA nanospheres loaded with silver nanoparticles with a controlled size able to inhibit bacterial growth were prepared, exploiting the reduction of silver nitrate by polydopamine spheres [21].

Both in situ and ex situ methods have been developed for incorporating AgNPs onto material surfaces [22]. The reducing catechol groups of PDA have further been explored with regard to their ability to form in situ well-dispersed AgNPs on different membranes with improved salt rejection and a maintenance of good permeability, simultaneously enhancing the membrane’s anti-adhesive and antimicrobial properties [23]. Polysulfone membranes were also similarly modified to mitigate biofouling [24]. PDA was used to form a thin layer and induce AgNP formation without the use of additional reductants or stabilizers on central venous catheters or poly(ether ketone) implants, achieving both significant antimicrobial efficacy and limited biological side effects [25,26,27].

Although the methods of preparation and application of PDA NPs are rapidly increasing, the structures and polymerization mechanisms used to obtain, purified, and define the size of NPs still represent an open issue, with limitations in their use [28]. While the development of PDA-based films is at a more advanced stage, there are no particular techniques or methods of synthesis in suspension able to yield nanoparticles with a controlled size, high efficiency, and high purity. Therefore, a purification step is required. This type of step is often vaguely defined and achieved mainly by centrifugation, not taking into account the coexistence of different species.

The development of a method for the deep, native characterization of these nanoparticles either during their synthesis or as final product represents another crucial point that must be addressed before these nanoproducts can be used as antimicrobial tools in medical applications. Size, polydispersity, and morphology are all parameters that directly impact nanoparticle activity (and toxicity), and their determination in their native state is of fundamental importance in understanding and predicting the potential of NPs.

Synthesized particles are usually characterized with FT-IR spectra for the identification of functional groups. Scanning and transmission electron microscopy (SEM, TEM) are among the most used techniques for the direct determination of the geometric size, shape, and ultrastructural properties of NPs [29].

However, the observed values may significantly differ from the native size/morphology that NPs display in liquid dispersion. Light scattering (LS) methods are also broadly used to analyze the size of NPs, and dynamic LS (DLS) is perhaps the most widely used technique for hydrodynamic size distribution analysis [30,31]. However, DLS gives no information on particle shape and density distribution and, in case of samples with complex, multimodal PSD, the accuracy of DLS-based PSD analysis may be intrinsically limited. Static, multi-angle LS (MALS) gives independent information on NP molar mass (M_r_) and root-mean-square (rms) radius values [32]. Consequently, it may provide information on the conformation and structure of NPs. As in the case of DLS, the detection accuracy of MALS is reduced for NPs with complex and multimodal PSD. These techniques are not suitable for directly giving precise information on dispersed nanoparticles, specifically when many species coexist and samples present a relatively high complexity and heterogeneity. The hyphenation of DLS or MALS detection to size-based separation methods can thus enhance the accuracy of the size analysis of complex NP samples.

The use of size-based separation techniques, such as field-flow fractionation (FFF), with spectroscopic and light scattering detection has already been shown as a unique platform able to characterize different NP in terms of their size, dimension, shape, and functional properties [33,34,35,36,37]. Recently, FFF was applied to the quantification of AgNPs in complex samples such as food samples and biological samples [38,39,40,41,42].

The micro-volume variant of FFF, hollow-fiber flow-field flow fractionation (HF5), was demonstrated to be capable of achieving a high performance and low dilution at the same time for the analysis of particles of different natures; this method also allows for applications where a disposable device is needed to avoid cross-contamination [43,44,45,46,47,48,49,50,51].

In HF5, separation is achieved without a stationary phase and by an external flow-field, perpendicular to the parabolic flow in an empty, porous capillary channel. The retention of NPs is inversely proportional to the hydrodynamic diffusion coefficient of the analyte and, consequently, to its M_r_ or hydrodynamic size. Selectivity is particularly high in the high-M_r_ and nanometer-size range [35]. HF5 coupled on-line with multiple detectors is able to separate and characterize populations such as proteins, colloids, polymers, and particulate materials up to about 100 μm in size. HF5 merges the advantages of being a miniaturized technique with the absence of the need for specific optimization for the separation of various nanoparticles depending on its sample properties, such as purity, density, solubility, hydrophobicity, solution conductivity, and particle isoelectric charge.

HF5 has already been applied to the deep characterization of silver nanoparticles, providing fundamental information on their size/morphological characterization and ion release quantification in various conditions (e.g., dilution, preservation media, coated surface) [50,52,53]. In this work, we present the use of an HF5-based analytical platform for the characterization of Ag-PDA NPs in dispersion, both during the synthesis steps to monitor particle formation and to characterize, separate, and collect purified particles. The goal is to provide a low-time, low-cost approach which can overcome the limitations of offline techniques and is able to integrate information about particle synthesis and formation; provide product characterization; and enable the prompt purification of isolated, biologically active nanoparticles. Indeed, the effectiveness of the purified AgPDA NPs in inhibiting bacterial growth was demonstrated in vitro against *S. aureus* and *E. coli* reference strains, as opposed to the lack of activity found for unpurified samples. The proposed approach can be used for synthesis optimization and for the quality control of the final AgPDA NPs product.

## 2. Materials and Methods

### 2.1. Reagents and Chemicals

All reagents were purchased from Sigma Aldrich (St. Louis, MO, USA). MilliQ water was employed for the mobile phase and synthesis medium.

### 2.2. Synthesis of Ag-PDA Nanoparticles

The synthesis of Ag-PDA nanoparticles was performed with an optimized method elaborated from the literature [52,53,54].

An initial screening of synthesis conditions was carried out to define the best reaction mixture (see Supplementary Materials: Figure S1 and Table S1), which was then used for this study. Briefly, ammonia aqueous solution (25%) was added to a mixture of ethanol in water (30% *v*/*v*). Following this, AgNO_3_ was added to achieve a final concentration of 0.5 mM. Finally, Dopamine-HCl was added drop by drop and the solution was kept under stirring. The final concentration of each species in the nanoparticle synthesis mix is reported in Table 1.

The stirring solution turned first yellow, indicating the beginning of AgNP formation [55], and then became black, indicating the beginning of the formation of polydopamine (melamin) composites. The sampling of the solution was performed during the color changes (15 min, 1 h, 2 h) and every two hours starting from 22 h to 30 h.

### 2.3. FFF-DAD-MALS

HF5 analyses were performed using an Agilent 1200 HPLC system (Agilent Technologies, Santa Clara, CA, USA) consisting of a degasser, an isocratic pump, and an Agilent 1100 DAD UV/Vis spectrophotometer combined with an Eclipse^®^ DUALTEC separation system (Wyatt Technology Europe, Dernbach, Germany). The system was connected to an 18-angle multiangle light scattering detector model DAWN HELEOS (Wyatt Technology Corporation, Santa Barbara, CA, USA). The HF5 cartridge (Wyatt Technology Europe) is commercially available and has a 10 kDa cutoff [56]. ChemStation version B.04.02 (Agilent Technologies) data system for Agilent instrumentation was used to set and control the instrumentation and for the computation of various separation parameters, complete with the Wyatt Eclipse @ ChemStation version 3.5.02 (Wyatt Technology Europe). ASTRA^®^ software version 6.1.7 (Wyatt Technology Corporation) was used to handle signals from the detectors (MALS and UV) and to compute the sample rg values.

After samples were injected in the HF fiber, the separation process was performed in four steps: (1) Focus. During focus, a flow of mobile phase was split into two different streams entering from inlet and outlet. This step was used to stabilize flows. (2) Focus–injection. The flow settings remained unvaried. The sample entered the channel through the inlet and the flow settings allowed us to focus analytes into a narrow band. (3) Elution. After sample injection, the flow settings changed and a flow of the mobile phase entered the channel only by the inlet. Part of it came out transversely through the channel pores (cross-flow), while the rest (channel flow, Vc) reached the detectors. The strength of the hydrodynamic file applied to nanoparticles to achieve their separation could be regulated by modifying the intensity of the cross-flow while analytes were eluted along the fiber towards the detectors. This parameter could be modified throughout the analysis to generate a decreasing cross-flow (namely, gradient). (4) Elution–injection. The cross-flow was set to zero and the mobile phase flowed along the fiber to the detectors, allowing for any remaining sample due to the cross-flow action inside the channel to be released. Additionally, the flow was redirected in the injection line to clean it before the next injection. The flow conditions for the HF5 method developed are detailed in Table 2, Where longitudinal flow is indicated by Vc and cross/focus flow as Vx.

Multi-angle light scattering (MALS) was used to calculate the gyration radius of eluting species, since it allows for the absolute determination of the particle root mean square radius of gyration (Rg) by measuring the net intensity of light scattered by such particles at a range of fixed angles. The polidispersity index of the two populations obtained from the FFF analysis was also obtained from the LS data. The separation method was used to characterize the synthesis and fractionate Ag-PDA nanoparticles. The injection volume was set to 10 μL for synthesis monitoring and 50 μL for particle characterization and collection. All analyses were carried out in Milliq water as the mobile phase. For antimicrobial evaluation, a non-separative, non-filtrating (flow injection analysis, FIA) and non-separative, filtrating (Focus-FIA) method were also employed. An FIA is a shortened, non-separative method: the sample is injected into the channel in the absence of cross/focus flow and reaches the detector without separation. This allows 100% of the sample to be collected. Instead, in a Focus-FIA, the sample components smaller than the membrane cutoff are filtered out and only the colloidal portion of the sample goes through the detector, while unreacted dopamine and Ag ions are removed.

### 2.4. SEM Analysis

For morphological examinations, a few drops of each sample of interest were deposited onto a metallic stub and allowed to dry. A Philips XL-20 scanning electron microscope operating at 15 kV was used for this. Samples were sputter-coated with gold before examination. Energy dispersive X-ray spectrometry (EDX) measurements were also performed to evidence the presence of silver.

### 2.5. X-ray Powder Diffraction Analysis

For X-ray investigations, a few drops of each sample of interest (FFF fractions 1, 2) were deposited onto a recessed silicon glass and allowed to dry. X-ray powder diffraction analyses were carried out by means of a Philips X’Celerator powder diffractometer equipped with a graphite monochromator in the diffracted beam. CuKα radiation (λ = 1.54 Å; 40 mA, 40 kV) was used. The 2θ range was from 30° to 80°, with a step size of 0.0668° and a scan rate of 500 s/step.

### 2.6. Antibacterial Activity

The antibacterial activity of the FIA, Focus-FIA, and method fractions was evaluated in vitro against *Staphylococcus aureus* (ATCC 25923) and *Escherichia coli* (ATCC 25922), which were selected as representative strains for Gram-positive and Gram-negative bacteria. The effectiveness of the samples in inhibiting bacterial growth was assessed by a standardized microdilution broth method using a 96-well plate [57]. Briefly, the bacterial suspensions were prepared at 0.5 McFarland, diluted 1:200 in Mueller–Hinton Broth (Sigma-Aldrich), and incubated with dilutions of each sample, starting from a ten-fold dilution of the volume collected from the fractionation process. Experiments included controls used to measure the bacterial growth in regular medium (positive control) and to check the background turbidity of the reagents and the sterility of the procedures (negative controls). The microplate was incubated at 37 °C and bacterial growth was monitored by measuring the Optical Density at 630 nm (Multiskan Ascent microplate reader, Thermo Fisher Scientific Inc., Waltham, MA, USA). Percentage values of samples in the different experimental conditions were determined relative to the positive growth control.

## 3. Results

The synthesis of pure, unmodified, and reliable nanoparticles is necessary to ensure their safe and standardized use; this is particularly true for clinical applications. In our work, we addressed the critical steps of the synthesis of a clinically relevant nanocomposite carrying both an antibacterial agent and a biocompatible material. The polymerization dynamics of dopamine are complex, time consuming, and often yield a mix of co-products. Likewise, the deposition of silver in the particulate is scarcely controlled and requires adequate method optimization and cleanup and thorough offline characterization. A series of post-synthesis steps is often performed, including cycles of ultracentrifugation and washing. However, these steps could modify the structure of the material; induce aggregation; and induce the release of silver, which is prone to dissolution [50,58].

Within this framework, we devised a separation method able to (1) monitor the synthesis of the nanocomposite in a TLC-like fashion and (2) provide the characterization and purification of isolated Ag-PDA using the same optimized separation method coupled to spectroscopic and size characterization. Indeed, the presence of non-destructive, orthogonal detectors, such as a DAD detector and multi-angle light scattering, offered the simultaneous size determination and identification of the different species obtained during the conjugation, and allowed the reaction to be carried out to completion, avoiding the waste of time and chemicals.

### 3.1. Monitoring the Synthesis of Ag-PDA

**Figure 1:**
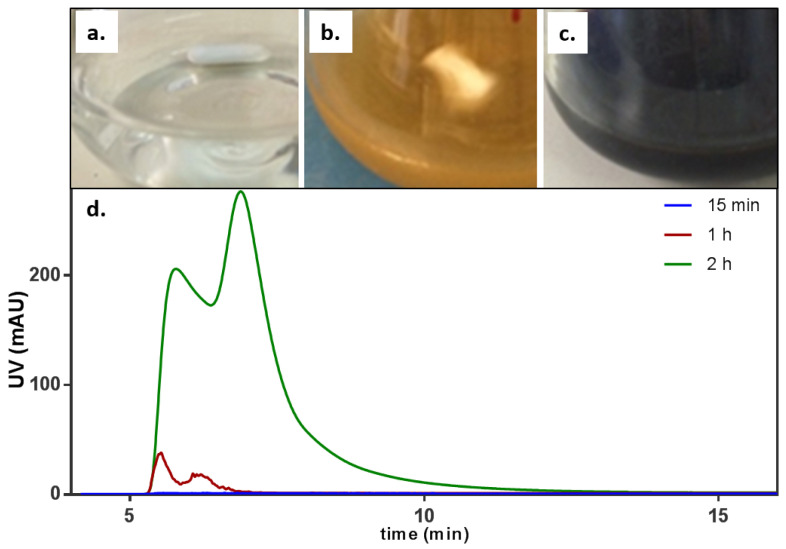
Appearance of the mixed solution over time after (**a**) 15 min, (**b**) 1 h, and (**c**) 2 h. (**d**) Absorption signal corresponding to the first stages of Ag-PDA formation, monitored by sampling and injection into FFF multidetection after 15 min (blue), 1 h (red), and 2 h (green).

Following dopamine addition to AgNO_3_ according to an optimized synthesis protocol (see Section 2.2 and Supporting Materials), the solution was followed during the initial phase (at 15 min, 1 h and 2 h) by collecting 10 μL of reaction medium each time and analyzing it via HF5 multidetection. The absorption profiles and the solution outlook are shown in Figure 1. It is clear how the color changed from transparent (Figure 1a) to yellow (Figure 1b) to black (Figure 1c), indicating both the initial formation of Ag seeds and the polymerization of dopamine. At the same time, the absorption signals obtained at 310 nm showed a band appearing and then increasing in intensity at a low retention time (5 min) and developing into a second band at 7 min. However, these species are still very small and barely retained by the system, since they are eluted at the void (5 min). The high absorption and corresponding flat signal of the laser scattering detection (not shown) indicates the lack of a nanodispersed sample.

**Figure 2:**
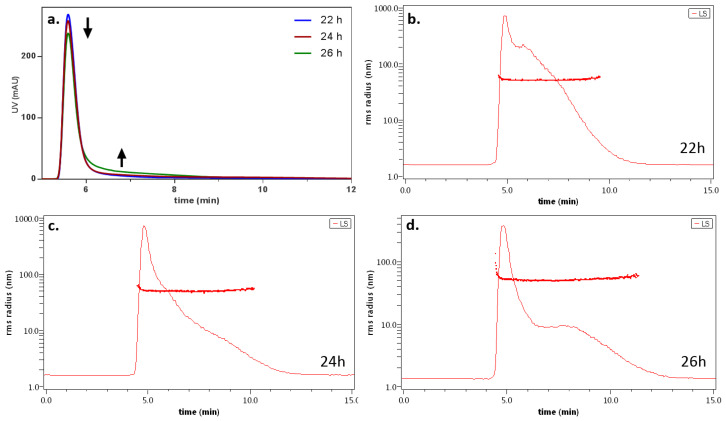
(**a**) Overlay of the UV absorption signal at 310 nm at three timepoints (22, 24, and 26 h). (**b**–**d**) LS signal (at 90°) and calculated radius of the species detected at the three timepoints.

Following this, the reaction was monitored until (i) a peak was visible and intense and (ii) the peak was also detectable from the MALS analysis, which occurred after a reaction time of about 20 h, which was consistent with the literature [54,55,56]. After this, the real-time analysis was performed every two hours and showed the process of formation of Ag-PDA nanoparticles (Figure 2).

In Figure 2a, the UV absorption at three timepoints (22, 24, 26 h) is overlaid: the intense band at 5 min is common for the three profiles, but becomes slightly lower with the increase in the reaction time; with the same trend, the peak tails with increasing absorption (black arrows). This behavior could go undetected using only UV detection, but the formation of a new, different species is made clear by the observation of the LS trace (Figure 2b–d). In fact, the light scattering signal is more sensible to size increases and allows us to observe the insurgence of a second species. Moreover, the gyration radius is calculated (overlaid on the panels), showing that the species under formation has a good monodispersion. The measured radii ranged from (38 ± 5) nm (at 22 h) to (45 ± 4) nm after 26 h; this is in line with the time retention shift of the emerging species, corresponding to particle growth. A similar dataset regarding Ag-PDA synthesis would be almost impossible to obtain by only relying on offline techniques such as imaging, requiring a more cost- and time-intensive experiment. On the other hand, “faster” techniques such as DLS and spectra acquisition would have been unable to identify the formation of different species, particularly in the early stages of particle formation.

### 3.2. Online Characterization of Ag-PDA

**Figure 3:**
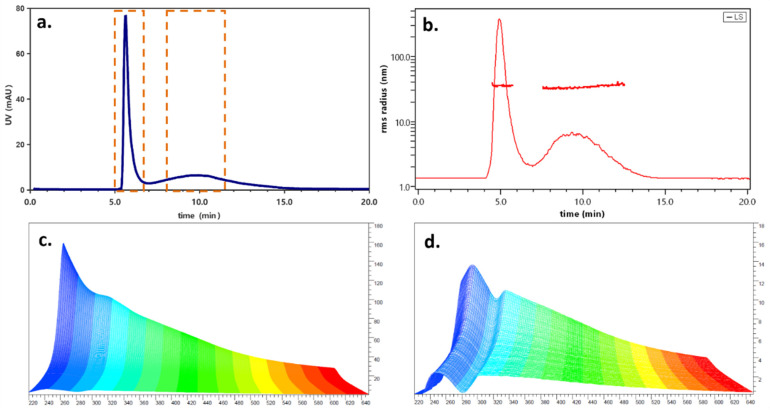
(**a**) UV fractogram at 310 nm; dashed selections: collection ranges. (**b**) Corresponding laser scattering profile. (**c**) UV spectrum of first band. (**d**) UV spectrum of second band.

The reaction was monitored until the relative intensity of the two bands reached a plateau. This was observed after a reaction time of 30 h, when the second band was also resolved from the first. At later times, no difference was observed, indicating that the two peaks detected (one at 5 min, the second at 10 min, dashed selections in Figure 3a) consisted of two distinct species likely reaching equilibrium.

The scattering profile (Figure 3b) is relatively more intense for the second band, which is compatible both with the increased hydrodynamical size and the presence of a stronger scatterer such as silver. The radius calculation also highlights the difference between the two species. The first is eluted at the void time and displays an unusually high gyration radius (35 ± 6 nm), which could be an artifact due to the presence of intertwined polymeric chains lacking an organized 3D structure. These chains could be eluted with an inverse mechanism due to their elongated morphology and could penetrate the parabolic flow by steric hindrance rather than diffusion [59]. This hypothesis is also supported by a high polydispersity index (PDI = 1.105), obtained from the LS data. A PDI value of 1.000 corresponds to a monodispersed sample. The second band instead had a gyration radius of (47 ± 5) nm. In order to verify the hydrodynamical size of Ag-PDA nanoparticles, a size standard (polystyrene beads, geometrical radius = 51 nm) was injected (not shown). The resulting retention time was found to be identical to that of band 2, indicating that the estimated rh of this species the same. In this case, the particles appeared to be monodispersed, with a PDI of 1.016.

Further insights into the morphology and arrangement of the PDA–silver nanocomposite can be obtained by studying its shape factor. This value, calculated as the rg/rh ratio, indicates where the mass of a particle is located with respect to its center of mass and is directly linked to morphology. Numerically, a shape factor of 0.8 is typical for solid spheres (such as polystyrene beads), increasing to 1 for hollow spheres and reaching higher values for more elongated structures such as rods (1.4) and random coils (1.6). A value closer to 1 is typical for either core-shell systems or prolate ellipsoids. The value found for particles from fraction 2, which is 0.9, can be justified by the presence of nanosilver localizing on the surface of the nanoparticle and creating a denser shell and by a more elongated structure than that of a regular sphere [60]. The size, shape, and polydispersity results obtained for the two species are shown in Table 3.

The two FFF-separated species were also different in terms of their spectroscopical behavior (Figure 3c,d). The first one had a spectrum typical for polydopamine, while the second displayed a local maximum at 390 nm, compatible with the presence of nanosilver embedded in the particle. This information is crucial for the intended use of such a material: indeed, the presence of a biocompatible polymer which could be deposited as a film would hinder the application of a silver delivery system, while the identification and separation of the two species would lead to the production of an effective antiseptic candidate [61]. Based on this evidence, two fractions were collected (one from min 4 to 6 and one in the range of 8.5–11.5 min) and subjected to further offline characterization.

### 3.3. Purification and Offline Characterization

**Figure 4:**
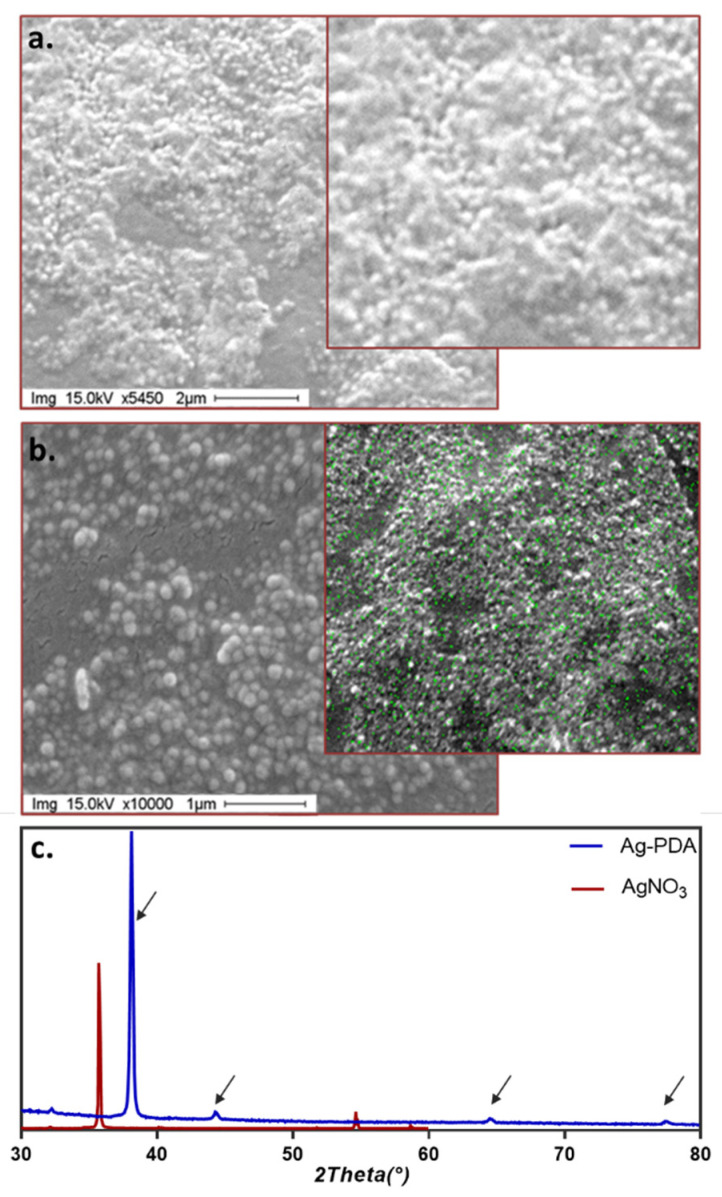
(**a**) SEM image of the first fraction (**b**) SEM image of the second fraction. Inset: EDX map. (**c**): X-rays diffraction patterns of the second fraction (blue line) collected (arrows: typical reflections of nanoAg), superimposed on the AgNO_3_ pattern as a control (red line).

The two fractions were imaged through scanning electron microscopy (SEM). The first species collected (Figure 4a) displayed a more amorphous structure, indicating the lack of a tridimensional organization, as expected from the results of the FFF characterization. The second fraction, in contrast, consisted of well-defined, spherical particles with a diameter of about 100 nm (Figure 4b).

Energy dispersive X-ray analysis (EDX) (Figure 4, insets) shows the presence of silver only for the second fraction (green points), as hypothesized from the absorption maximum observed in Figure 3d. If batch characterization would have left room for speculation about the presence of free silver in the suspension due to the presence of unreacted AgNO_3_ or silver nanoparticles alone, the use of FFF separation and purification removes all doubts. In fact, the fractionation process also includes a filtration step (i.e., focusing; see M&M), which would eliminate free ions present in the reaction mix, and only preserve Ag if the metal took part in the nanostructure.

While the presence of silver in the nanostructure was confirmed, it was necessary to determine the nature of silver in the second fraction. This was achieved by X-ray analysis, which is shown in Figure 4c together with the spectrum of AgNO_3_ as a reference.

The results confirm the presence of metallic nano silver, indicating that the sample corresponds to purified, functionalized Ag-PDA nanoparticles. Indeed, the corresponding powder X-ray diffraction pattern shows characteristic peaks of Ag crystal found at 38.092, 44.214, 64.409, and 77.344 on the 2θ scale, corresponding, respectively, to the (1 1 1), (2 0 0), (2 2 0), and (3 1 1) crystal planes. There was no signal corresponding to Ag^+^.

### 3.4. Antibacterial Activity

**Figure 5:**
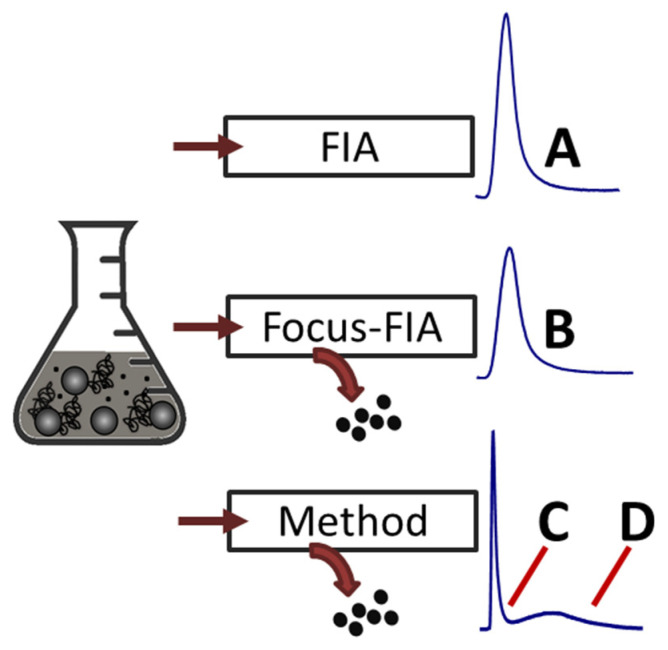
Schematization of the FFF fraction collection approach used to obtain comparable fractions of whole sample (**A**), filtered sample (**B**), polymeric PDA (**C**), and Ag-PDA (**D**). Dots represent small molecules and Ag ions.

In order to verify the antimicrobial activity of the freshly synthesized Ag-PDA nanoparticles and evaluate the effect of the FFF purification, a set of samples was produced to represent the reaction mix: free dopamine and potential unreacted Ag^+^ ions (A), the colloidal portion made of both polymeric PDA and Ag-PDA particles (B), the purified polymeric population (C) and the purified Ag-PDA particles (D). This was achieved by the collection of HF5 fractions via two non-separative methods (FIA and Focus-FIA, as described in Section 2.3) and the previously employed separation method. The isolation process is schematized in Figure 5.

The collected samples A–D were diluted to the same final concentration, as calculated from their relative area at 300 nm, and subjected to antibacterial activity tests.

**Figure 6:**
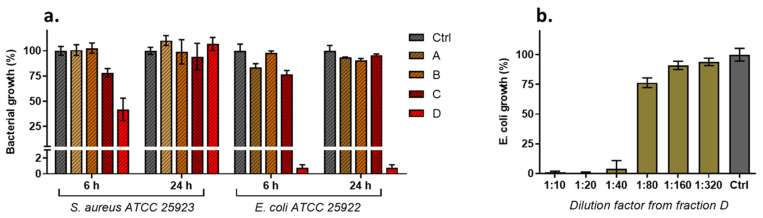
(**a**) Antibacterial activity of the samples (ten-fold diluted in MH broth) against *S. aureus* (left) and *E. coli* (right) reference strains after 6 and 24 h of incubation. Data are expressed as percentage values of the bacterial growth relative to the positive control. (**b**) Antibacterial activity of fraction D (serial two-fold dilutions) against *E. coli* reference strain following 24 h of incubation.

The inhibitory activity of the different fractions was assayed in vitro against both Gram-positive and Gram-negative bacteria by a broth microdilution method. Bacterial growth of *S. aureus* and *E. coli* was monitored at different times, and the results demonstrated that the only biologically active fraction was D, consisting of purified Ag-PDA nanoparticles, as reported in Figure 6a. The lack of the activity of fraction C, where only polydopamine is present (as confirmed both by online and offline characterization), was also confirmed, as expected. Interestingly, fractions A and B, where Ag is present both as an ion and as unpurified Ag-PDA nanoparticles, were found to be ineffective. This could be explained by the presence of polydopamine in the suspension, which hinders interaction between nanosilver and bacterial cell walls, confirming the necessity of particle purification.

Growth data analysis showed that Gram-negative *E. coli* (Figure 6a, left) had a higher sensitivity than Gram-positive *S. aureus* (Figure 6a, right). Indeed, the cell wall structure plays a crucial role in the antibacterial activity of Ag-based compounds; it is well known that Gram-negative bacteria are more susceptible due to their narrower cellular walls compared to Gram-positive strains [62]. The purified Ag-PDA nanoparticles in fraction D completely inhibited *E. coli* when used at the highest dilution (1:10 with respect to the volume collected from the fractionation process) and with up to 24 h of incubation.

Given this result, serial two-fold dilutions of fraction D were also assayed against *E. coli.* The results confirmed the effectiveness of the sample in inhibiting bacterial growth and highlighted the specific interaction of the HF5-isolated Ag-PDA nanoparticles with bacterial membranes, leading to dose-dependent activity (Figure 6b).

## 4. Conclusions

In our work, we addressed the critical steps of the synthesis of a clinically relevant nanocomposite carrying both an antibacterial agent (nanosilver) and a biocompatible material (polydopamine), Ag-PDA. Given the complexity of the synthesis mechanisms and the difficulty of controlling its process, a series of post-synthesis steps is often performed after this process, including cycles of centrifugation and washing. However, these steps can be ineffective, modify the structure of the material, induce aggregation, and induce the release or dissolution of silver.

Within this framework, we devised a fast, non-destructive method able to (1) monitor the synthesis of the nanocomposite in a TLC-like fashion and (2) Provide the characterization and purification of isolated Ag-PDA using the same, optimized separation method based on miniaturized FFF coupled to spectroscopical and size characterization. Orthogonal UV and light scattering detectors offered the simultaneous size determination and identification of the different species obtained during the conjugation, which were confirmed through conventional, offline characterization. The comparison between unfractionated, filtered, and isolated nanoparticles confirmed the antibacterial effectiveness of Ag-PDA, especially towards Gram-negative *E. coli*, but also highlighted how the isolation of these particles proved crucial to avoid interference and the loss of activity. The FFF multidetection approach allowed the reaction to be carried out to completion, avoiding the waste of time and chemicals; allowed fast on-line characterization; and provided isolated, purified, and biologically active Ag-PDA particles in water, ensuring their safe use as biocompatible antibacterials.

## Figures and Tables

**Table 1:** Concentration of reagents and medium used in Ag-PDA synthesis.

Ag (AgNO_3_) (mM)	Dopamine-HCl (mM)	EtOH (% *v*/*v*)	Ammonia (% *v*/*v*)
0.5	10	30	1

**Table 2:** Flow conditions used for the HF5 analyses of PDA and Ag@PDA.

Focus (mL/min)	Focus-Injection (mL/min)	Elution (mL/min)	Elution-Inject (mL/min)
Vx = 0.8 T = 2 min	Vx = 0.8 T = 3 min	Vx = 0.10 T = 20 min	Vx = 0.00 T = 6 min

**Table 3:** Summary of the size and morphology information of the two populations separated and characterized through FFF multidetection.

	Hydrodynamic Radius (nm)	Gyration Radius (nm)	PDI	Shape Factor
Species 1	-	35 ± 6	1.109	>>1 *
Species 2	51	47 ± 5	1.016	0.9

* apparent.

## Supplementary Materials

The following supporting information can be downloaded at https://www.mdpi.com/article/10.3390/antibiotics11030358/s1. Figure S1: Solid line: LS profile of Ag-PDA particles obtained with R1-R2-R4-R5 Ag/DA ratios. Dotted dis-tribution: Gyration radii; Table S1: concentration of reagents and medium used in Ag-PDA synthesis.
