# Supplementary material for: Synthesis Monitoring, Characterization and Cleanup of Ag-Polydopamine Nanoparticles Used as Antibacterial Agents with Field-Flow Fractionation
Source: Antibiotics (Basel). 2022 Mar 8;11(3):358. doi: 10.3390/antibiotics11030358 (PMC8944547; doi:10.3390/antibiotics11030358)
Supplement: Supplementary file 1 [file antibiotics-11-00358-s001.zip › antibiotics-1624639-supplementary.pdf]

antibiotics

Article

Synthesis Monitoring, Characterization and Cleanup of Ag-Polydopamine Nanoparticles Used as Antibacterial Agents with Field-Flow Fractionation

Valentina Marassi [1,2,*], Sonia Casolari [1], Silvia Panzavolta [1], Francesca Bonvicini [3], Giovanna Angela Gentilomi [3], Stefano Giordani [1], Andrea Zattoni [1,2], Pierluigi Reschiglian [1,2] and Barbara Roda [1,2]

Supplementary Materials

Table S1. concentration of reagents and medium used in Ag-PDA synthesis.

	Ag (AgNO3) (mM)	Dopamine-HCl (mM)	EtOH (% v/v)	Ammonia (% v/v)
R1	2	10	30	1
R2	1	10	30	1
R3	0.5	10	30	1
R4	0.5	2.5	30	1
R5	1	2.5	30	1

The synthesis of Ag-PDA nanoparticles was performed with an optimized method elaborated from literature [54-56, manuscript]. The most promising reagent ratio (green) was chosen from a screening of different Ag/Dopamine ratios shown in Table S1.

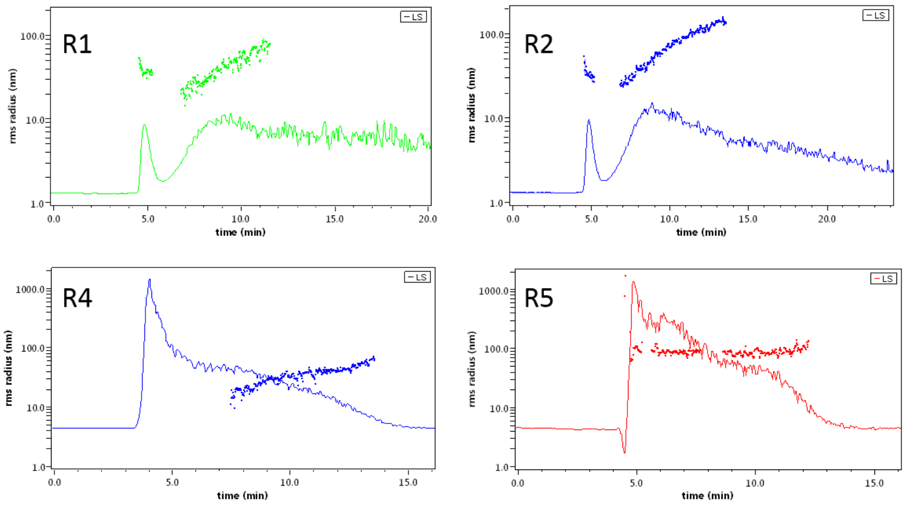

Figure S1. Solid line: LS profile of Ag-PDA particles obtained with R1-R2-R4-R5 Ag/DA ratios. Dotted distribution: Gyration radii.

Each reaction (R1-R5) was carried out for 30 hours. A 50µL aliquot was injected and analysed with FFF-multidetection to verify the formation of Ag-PDA nanoparticles.

The results showed that an increase in Ag concentration yielded less monodispersed particles, ranging from 25 to >100 nm in terms of Gyration radius (Figure S1, R1-R2).

A lower dopamine concentration led to a scarce resolution from the first population, suggesting that the formation of PDA particles was less effective. (Figure S1, R4-R5). R3 was then chosen for time monitoring, online and offline characterization, and activity tests.
